# CCl_4_-induced hepatotoxicity: protective effect of rutin on p53, CYP2E1 and the antioxidative status in rat

**DOI:** 10.1186/1472-6882-12-178

**Published:** 2012-10-08

**Authors:** Rahmat A Khan, Muhammad R Khan, Sumaira Sahreen

**Affiliations:** 1 Department of Biotechnology, Faculty of Biological Sciences, University of Science and Technology Bannu, Khyber Pakhtunkhwa, Pakistan; 2 Department of Biochemistry, Faculty of Biological Sciences, Quaid-I-Azam University Islamabad, Islamabad, Pakistan; 3 Botanical Science Divisions, Pakistan Museum of Natural History, Islamabad, Pakistan

**Keywords:** Hepatotoxicity, Rutin, p53, CYP 2E1, Antioxidant enzymes

## Abstract

**Background:**

Rutin is a polyphenolic natural flavonoid which possesses antioxidant and anticancer activity. In the present study the hepatoprotective effect of rutin was evaluated against carbon tetrachloride (CCl_4_)-induced liver injuries in rats.

**Methods and materials:**

24 Sprague–Dawley male rats were equally divided into 4 groups for the assessment of hepatoprotective potential of rutin. Rats of group I (control) received only vehicles; 1 ml/kg bw of saline (0.85%) and olive oil (3 ml/kg) and had free access to food and water. Rats of group II, III and IV were treated with CCl_4_ (30% in olive oil, 3 ml/kg bw) via the intraperitoneal route twice a week for four weeks. The rutin at the doses of 50 and 70 mg/kg were administered intragastrically after 48 h of CCl_4_ treatment to group III and IV, respectively. Protective effect of rutin on serum enzyme level, lipid profile, activities of antioxidant enzymes and molecular markers were calculated in CCl_4_-induced hepatotoxicity in rat.

**Results:**

Rutin showed significant protection with the depletion of alanine aminotransferase (ALT), aspartate aminotransferase (AST), alkaline phosphatase (ALP), gamma glutamyl transpeptidase (γ-GT) in serum as was raised by the induction of CCl_4_. Concentration of serum triglycerides, total cholesterol and low density lipoproteins was increased while high-density lipoprotein was decreased with rutin in a dose dependent manner. Activity level of endogenous liver antioxidant enzymes; catalase (CAT), superoxide dismutase (SOD), glutathione peroxidase (GSHpx), glutathione-S-transferase (GST) and glutathione reductase (GSR) and glutathione (GSH) contents were increased while lipid peroxidation (TBARS) was decreased dose dependently with rutin. Moreover, increase in DNA fragmentation and oxo8dG damages while decrease in p53 and CYP 2E1 expression induced with CCl_4_ was restored with the treatment of rutin.

**Conclusion:**

From these results, it is suggested that rutin possesses hepatoprotective properties.

## Background

Exposure to toxic chemicals, environmental pollutants and drugs can cause cellular injuries through metabolic activation of reactive oxygen species (ROS) [1]. Carbon tetrachloride (CCl_4_) has been used extensively to study hepatotoxicity in animal models by initiating lipid peroxidation, thereby causing injuries to kidney, heart, testis and brain [2-4], in addition to liver pathogenesis [5]. Liver is particularly susceptible to oxidative stress due to the direct release of CCl_4_ metabolites and cytokines, which propagate inflammatory response [6]. CCl_4_ is one of the xenobiotics that has been reported to induce acute and chronic tissue injuries [7,8] through bioactivation of the phase I cytochrome P450 system to form reactive metabolic trichloromethyl radicals (·CCl_3_) and peroxy trichloromethyl radicals (·OOCCl_3_). These free radicals can covalently bind to macromolecules such as proteins, lipids and nucleic acids. The double allylic hydrogen bonds of polyunsaturated fatty acid (PUFA) are susceptible to abstraction by free radicals; CCl_4_ exposure induces an increase in lipoperoxide and free peroxide radical concentrations that are highly reactive and cause injury or necrosis [9,10].

An increase in unsaturated fatty acid lipoperoxide and free peroxide radical concentrations [11,12], can induce alterations in the cholesterol profile and decrease in hepatic antioxidant enzymes [13], in addition to induction of oxidative DNA damage including formation of DNA adducts, genetic mutations, strand breakage and chromosomal alterations [14]. These free radicals can cause depletion of CYP2E1 activity [15] and increase in oxo8dG concentration in tissues of experimental animals [16]. DNA fragmentation induces *p53* gene expression, blocks cells in the G phase of the cell cycle, and gives additional time for DNA repair, while severe DNA damage triggers apoptosis [17]. It has been reported that CCl_4_ administration increases the silver-stained nucleolar organizer region, alters its size, morphology or spreading in the nucleus, which may be utilized as an indicator of genotoxicity, neoplasia and hyperplasia to complement other histological procedures [11].

Flavonoids are a large group of polyphenolic compounds that play an important role in detoxification of free radicals and are markedly found in fruits, vegetables and medicinal plants [18,19]. Glycosidic flavonoids such as rutin are much more readily absorbed by humans than aglycones [20,21]. Rutin possesses antitumor [22], anti-inflammatory [23] and antimutagenic potential [24], besides myocardial protection [25] and immunomodulating activities [26]. Therefore, the present study was designed to investigate the hepatoprotective effects of rutin against CCl_4_-induced oxidative stress and its role in alleviation of lipid peroxidation and restoration of p53 and CYP2E1 activity.

## Methods

### Drugs and chemicals

Reduced glutathione (GSH), oxidized glutathione (GSSG), glutathione reductase, gamma-glutamyl p-nitroanilide, glycylglycine, bovine serum albumin (BSA), 1,2-dithio-bis nitro benzoic acid (DTNB), 1-chloro-2,4-dinitrobenzene (CDNB), reduced nicotinamide adenine dinucleotide phosphate (NADPH), rutin, CCl_4_, flavine adenine dinucleotide (FAD), glucose-6-phosphate, Tween-20, 2,6-dichlorophenolindophenol, thiobarbituric acid (TBA), picric acid, sodium tungstate, sodium hydroxide, trichloroacetic acid (TCA) and perchloric acid (PCA) were purchased from Sigma Chemicals Co. USA.

### Animals and treatment

Six week old, 24 Sprague–Dawley male rats (200–210 g) were provided by National Institute of Health Islamabad and were kept in ordinary cages at room temperature of 25±3°C with a 12 h dark/light cycles. They have free access to standard laboratory feed and water, according to the study protocol approved by Ethical Committee of Quaid-i-Azam University Islamabad for animal care and experimentation. To study the hepatoprotective effects of rutin, rats were equally divided into four groups (six rats). Animals of group I were treated with 1 ml/kg bw of saline (0.85%) intragastrically and olive oil (3 ml/kg bw) intraperitoneally twice a week for four weeks. Rats of group II, III and IV were treated with CCl_4_ (30% in olive oil) at a dose of 3 ml/kg bw intraperitoneally twice a week for four weeks. Animals of group II received only CCl_4_ treatment. However, animals of group III and IV received rutin at a dose of 50 and 70 mg/kg bw intragastrically, respectively, in addition to CCl_4_ treatment, twice a week for four weeks.

After 24 h of the last treatment, all the animals were weighted, sacrificed, collected the blood while liver was removed, weighted and perfuse in ice-cold saline solution. Liver samples were treated with liquid nitrogen and stored at −70 °C for further studies.

### Assessment of hepatotoxicity

Liver marker enzymes (alanine aminotransferase (ALT), aspartate aminotransferase (AST), alkaline phosphatase (ALP), gamma glutamyl transpeptidase (γ-GT), lipid profile [total cholesterol, low-density lipoprotein (LDL), high-density lipoprotein (HDL) and triglyceride] were estimated by using standard AMP diagnostic kits (Stattogger Strasse 31b 8045 Graz, Austria). CYP 2E1 (cat no: E90988Ra, Uscn Life Science Inc.), oxo8dG and p53 (PUMA ELISA Kit cat no: E91909Ra) concentration was determined with ELISA kit.

### Assessment of oxidative stress

For determination of oxidative stress liver tissue was homogenized in 10 volumes of 100 mmol KH_2_PO_4_ buffer containing 1 mmol EDTA (pH 7.4) and centrifuged at 12,000 × g for 30 min at 4 °C. The supernatant was collected and used for the determination of protein and enzymatic studies as described below. Protein concentration was determined by using crystalline BSA as standard. CAT and SOD activities are determined with protocol of [27,28] while phase II metabolizing enzyme, including glutathione-S-transferase (GST), glutathione reductase (GSR), glutathione peroxidase (GSH-Px), reduced glutathione (GSH) [29-32] and thiobarbituric acid reactive substances (TBARS) contents, respectively [33].

### DNA damages

Hepatic DNA damages (fragmentation % by DPA assay), DNA ladder assay [34] and number of NORs per cell [35] were determined.

### Statistical analysis

To determine the treatment effects, one-way analysis of variance was carried by computer software SPSS 13.0. Level of significance among the various treatments was determined by LSD at 0.05% and 0.01% level of probability.

## Results

### Body weight, liver weight

Treatment of CCl_4_ caused significant reduction *(P<*0.01*)* in body weight while increased the absolute liver and relative liver weight comparatively to control group was significantly *(P<*0.01*)* restored with 50 mg/kg bw and 70 mg/kg bw treatment of rutin (Table 1).

**Table 1:** Effect of Rutin on absolute liver weight, relative liver weight, and % increase in body weight

**Treatment**	**% increase in body weight**	**Absolute liver weight (g)**	**Relative liver weight (% to body weight)**
Control	35.00±0.87++	7.00±0.23++	0.072±0.02++
3 ml/kg CCl_4_	24.52±0.92**	8.62±.89**	0.086±0.01**
50 mg/kg Rutin+CCl_4_	30.85±0.96++	7.21±0.15++	0.072±0.01++
70 mg/kg Rutin+CCl_4_	34.09±0.85++	7.19±0.17++	0.079±0.03++

Mean ±SE (n=6 number).

** indicate significance from the control group at *P<*0.01 probability level.

++ indicate significance from the CCl_4_ group at *P<*0.01 probability level.

### Lipids profile

Administration of CCl_4_ increased triglycerides, total cholesterol, LDL while decreased the HDL as shown in Table 2. Concentration of HDL was significantly (P<0.01) increased by rutin whereas concentration of triglycerides, total cholesterol and LDL was appreciably *(P<*0.01*)* augmented to compensate the CCl_4_-induced toxicity.

**Table 2:** Effect of Rutin on Lipids profile

**Treatment**	**Triglycerides (mg/dl)**	**Total cholesterol (mg/dl)**	**High density lipoprotein (mg/dl)**	**Low density lipoprotein(mg/dl)**
Control	7.82±0.45++	6.13±0.25++	3.62±0.21++	2.48±0.32++
3 ml/kg CCl_4_	11.13±0.58**	11.22±0.23**	2.83±0.18**	8.42±0.17**
50 mg/kg Rutin+CCl_4_	8.51±0.44++	5.72±0.20++	3.24±0.23++	2.52±0.28++
70 mg/kg Rutin+CCl_4_	7.93±0.90++	6.94±0.52++	3.82±0.28++	2.68±0.29++

Mean ±SE (n=6 number).

** indicate significance from the control group at *P<*0.01 probability level.

++ indicate significance from the CCl_4_ group at *P<*0.01 probability level.

### Genotoxicity studies

**Figure 1:**
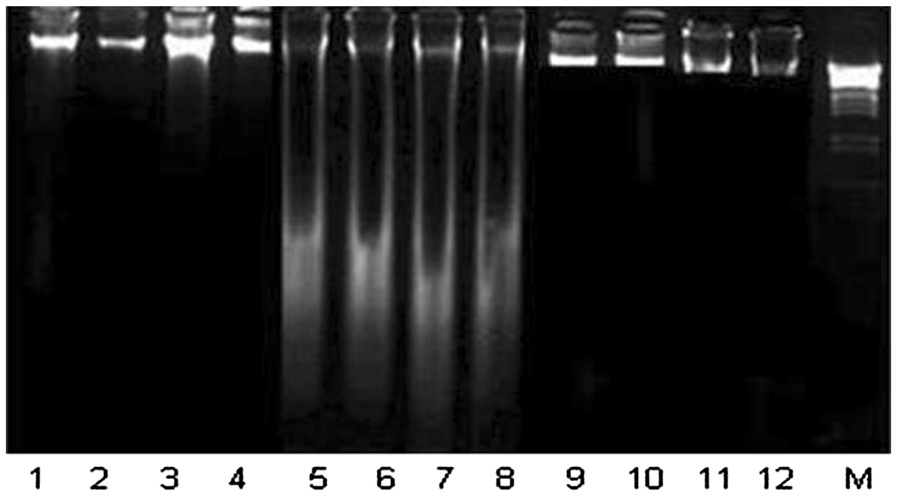
**Protective effects of rutin on DNA; Lane 1–4 (non treated control), 5–8 (CCl**_**4**_**treated rats), 9,10 (CCl**_**4**_**+50 mg/kg b. w. rutin), 11,12 (CCl**_**4**_**+70 mg/kg b.w. rutin).**

Exposure of CCl_4_ elicited the hepatic DNA damages (% fragmentation) and number of AgNORs/cell. Percent serum level of oxo8dG was increased whereas the % activity level of p53 and CYP 2E1 was decreased in hepatic samples of rat. Treatment of rats with 50 mg/kg bw and 70 mg/kg bw of rutin restored the level of these markers (Table 3). DNA ladder assay showed conformity to the DNA fragmentation assay (Figure 1).

**Table 3:** Effect of Rutin on Genotoxicity studies

**Treatment**	**AgNORS (NORs/cell)**	**%DNA fragmentation**	**% oxo8dG**	**% p53**	**% CYP 2E1**
Control	2.02±0.33++	5.33±1.46++	4.10±0.53++	7.13±0.63++	15.23±1.09++
3 ml/kg CCl_4_	6.42±0.29**	22.50±3.68**	11.20±0.27**	3.41±0.49**	8.10±0.78**
50 mg/kg Rutin+CCl_4_	3.32±0.76++	7.61±1.04++	6.13±0.26++	5.15±0.46++	12.16±1.04++
70 mg/kg Rutin+CCl_4_	3.11±0.35++	5.00±1.83++	5.20±0.85++	7.10±0.55++	14.22±1.33++

Mean ±SE (n=6 number).

** indicate significance from the control group at *P<*0.01 probability level.

++ indicate significance from the CCl_4_ group at *P<*0.01 probability level.

### Indices of hepatotoxicity

Administration of CCl_4_ markedly increased (P<0.01) the activity of liver serum marker enzymes such as AST, ALT, ALP and γ-GT as compared with the control group. Elevations in the secretion of these enzymes were significantly decreased (P<0.01) by 50 mg/kg bw and 70 mg/kg bw of rutin as compared to the CCl_4_ group are shown in Table 4.

**Table 4:** Effect of Rutin on Indices of hepatotoxicity

**Treatment**	**ALT (U/L)**	**AST (U/L)**	**ALP (U/L)**	**γ-GT (nM/min /mg protein)**
Control	52.11±2.12++	76.32±2.74++	198.10±3.93++	105.25±2.22++
3 ml/kg CCl_4_	111.01±3.42**	234.10±4.27**	470.13±6.49**	154.03±3.02**
50 mg/kg Rutin+CCl_4_	58.01±2.68++	102.13±4.99++	227.05±3.89++	111.03±3.04++
70 mg/kg Rutin+CCl_4_	56.12±2.05++	98.61±2.75++	209.06±3.68++	107.23±2.01++

Mean ±SE (n=6 number).

** indicate significance from the control group at *P<*0.01 probability level.

++ indicate significance from the CCl_4_ group at *P<*0.01 probability level.

### Assessment of oxidative stress

CCl_4_ treatment in rats significantly decreased *(P<*0.01*)* the activity of CAT, SOD, GST, GSH-Px, GSR, GSH while increased TBARS contents in liver samples. The increase of lipid peroxidation caused; reduction in the activities of antioxidant enzymes and glutathione (GSH) contents were markedly attenuated *(P<*0.01*)* by administration of 50 mg/kg bw and 70 mg/kg bw of rutin in intoxicated rats (Table 5).

**Table 5:** Effect of Rutin on assessment of oxidative stress

**Treatment**	**CAT (U/min)**	**SOD (U/mg protein)**	**GST nM /min/mg protein**	**GSH-Px nM/min/mg protein**	**GSR nM /min/mg protein**	**GSH (μM /g tissue)**	**TBARS (nM /min/mg protein)**
Control	4.81±0.10++	22.71±1.18++	192.12±2.49++	84.12±5.19++	127.23±10.52++	1.23±0.02++	28.33±1.12++
3 ml/kg CCl_4_	2.91±0.06**	11.91±0.38**	104.23±2.14**	46.12±3.62**	62.33±9.28**	0.60±.01**	52.17±2.61**
50 mg/kg Rutin+CCl_4_	4.60±0.16++	19.52±0.47++	175.21±2.19++	70.23±4.31++	101.13±9.14++	1.17±0.04++	31.17±1.74++
70 mg/kg Rutin+CCl_4_	5.03±0.09++	21.41±0.62++	183.32±3.09++	80.12±6.82++	121.12±12.24++	1.18±0.01++	27.14±1.22++

Mean ±SE (n=6 number).

** indicate significance from the control group at *P<*0.01 probability level.

++ indicate significance from the CCl_4_ group at *P<*0.01 probability level.

## Discussion

The fields of dietary modification and chemoprevention show considerable effective approaches against oxidative stress and are the focus of research these days [36]. Various studies have shown that several mutagens and carcinogens cause generation of oxygen-free radicals, which play a major role in the emergence of cancer and other health disturbances [37,38]. The present study revealed that CCl_4_-induction in rats remarkably increased the level of ALT, AST, ALP and γ-GT. CCl_4_ causes acute hepatocyte injuries, altered membrane integrity and as a result enzymes in hepatocytes leak out [39]. However, after treatment with rutin, the pathological increases in ALT, AST, ALP and γ-GT were significantly restored. These results indicate that rutin has the ability to protect against CCl_4_-induced hepatocyte injury, which is in agreement with a previous study [40] that reported the protective consequence of polyphenolic compounds against CCl_4_-induced liver cirrhosis. Importantly, the increased serum concentrations of triglycerides, total cholesterol and LDL, and the decreased level of HDL, were restored to normal values with rutin co-treatment*.* This may be explained on the basis that rutin has a strong ability to chelate multivalent metal ions, especially zinc, calcium and iron. Indeed, its ability to chelate minerals has been reported to have some protective effects, such as decreasing iron mediated free radical formation and lowering serum cholesterol, triglycerides and lipid peroxides in experimental animals [41]*.* Similar findings were reported in another study [42] that investigated the hepatoprotective effects of plant bioactive compounds against CCl_4_-induced hepatic injury in rats.

ROS formed during the biotransformation process of CCl_4_ are more reactive and toxic than the parental compound. Biotransformation of CCl_4_ occurs in the endoplasmic reticulum and the isoenzyme implicated in this process is CYP2E1 [43,44]. Our results showed that the active free radical/intermediate of CCl_4_ caused a reduction in CYP2E1, which was markedly restored by rutin treatment. Our results showed conformity with previous investigations, which demonstrated that the polyphenolic natural product is responsible for its protective, effects [45,46].

Results of the present study revealed that exposure of rats to CCl_4_ resulted in depletion of antioxidant activities. In consonance with our results, Szymonik-Lesiuk *et al.*[1] reported that CCl_4_ intoxication leads to changes in antioxidant enzymes and reactive intermediates involved in the bioactivation of CCl_4_ that may truss to those enzymes to prevent their inactivation. Furthermore, our results correspond with [11,12], and are in agreement with an investigation following CCl_4_ intoxication [47].

Glutathione provides a first line of defense and scavenges free radical oxygen species (ROS). The decreased concentration of GSH in liver may be due to NADPH reduction or GSH utilization in the exclusion of peroxides [48]. GSH-dependent enzymes offer a second line of protection as they primarily detoxify noxious byproducts generated by ROS and help to avert dissemination of free radicals [49]. GSH-Px detoxifies peroxides by reacting with GSH and converting it into GSSG, which is reduced to GSH by GSR [50]. Our study revealed that CCl_4_ treatment in rats markedly changed the activity of antioxidant enzymes, which was reverted by the co-administration of rutin. Thiobarbituric acid reactive substances (TBARS), the final metabolites of peroxidized polyunsaturated fatty acids, are considered as a late biomarker of oxidative stress [51]. In our experiments, major decrease in lipid peroxidation and consequent reduction in TBARS were obtained by treatment with rutin. The increment in lipid peroxidation, as assessed by the elevated levels of TBARS following CCl_4_ administration, has been well documented [12]. Data of the present study indicated that lipid peroxidation induced by oxidative stress caused DNA damage. TBARS react with the DNA strand to form the M_1_G adduct, the mutagenic pirimedopurinone adduct of deoxyguanosine [52]. Administration of rutin markedly reduced the DNA damage, which is in close agreement with a previous study [11]. This level of DNA damage decreases the expression of p53 and blocks cells in the G phase of the cell cycle, which gives the cells additional time to repair the DNA damage. However, severe DNA damage may elicit apoptosis [53]. The data revealed that CCl_4_-induction caused marked reduction in p53. This result may be explained on the basis that CCl_4_ acts as a tumor promoter through increasing the intracellular concentration of ROS necrosis/regeneration and cell proliferation and/or may be due to mutation of p53. Our results regarding p53 are in agreement with previous studies [54,55].

## Conclusion

These results demonstrate that administration of rutin may be useful in the treatment and prevention of hepatic stress.

## Competing interests

The authors declare that they have no competing interests.

## Authors’ contributions

**RAK** made a significant contribution to conception and design of the study, acquisition and analyses of data and drafting of the manuscript. **MRK** and **SS** made contribution in sample collection and design. All the authors read the revised manuscript and approved.

## Pre-publication history

The pre-publication history for this paper can be accessed here:

http://www.biomedcentral.com/1472-6882/12/178/prepub
